# Characteristics of COVID-19-Related Free Telephone Consultations by Public Health Nurses in Japan: A Retrospective Study

**DOI:** 10.3390/healthcare9081022

**Published:** 2021-08-09

**Authors:** Yuka Sumikawa, Chikako Honda, Kyoko Yoshioka-Maeda, Noriko Yamamoto-Mitani

**Affiliations:** 1 Department of Community Health Nursing, Division of Health Sciences and Nursing, Graduate School of Medicine, The University of Tokyo, Tokyo 113-0033, Japan; hchika-tky@g.ecc.u-tokyo.ac.jp (C.H.); noriko-tky@g.ecc.u-tokyo.ac.jp (N.Y.-M.); 2 Department of Health Promotion, National Institute of Public Health, Saitama 351-0197, Japan; yoshioka.k.aa@niph.go.jp

**Keywords:** community, COVID-19, public health nurse, telephone consultation

## Abstract

This study aimed to (1) describe the characteristics of community residents who used coronavirus disease 2019 (COVID-19)-related telephone consultation systems by public health nurses (PHNs) and (2) analyze the concerns they had during the first wave of COVID-19 in Japan. Among 1126 telephone consultations, PHNs recorded telephone consultations between 25 March, and 30 April, 2020, in City A, Japan. We analyzed 1017 consultations involving 799 (79%) community residents (resident group) and 218 (21%) organizational representatives (organization group) located in City A. Half of the consultations were made during midmorning, and most of the consultations were regarding COVID-19 symptoms. Among the resident group, visiting a primary care doctor was the most common recommendation by the PHNs; there was no difference in provision of consultation by sex. Health- and welfare-related organizations mainly consulted PHNs about “having COVID-19-related symptoms” and “undergoing PCR testing,” and PHNs’ recommended them to visit a primary care doctor and coordinate PCR testing. The results suggest that public health centers should provide more helpful information on COVID-19 that accurately reflects the concerns of the population.

## 1. Introduction

The spread of coronavirus disease 2019 (COVID-19) has gradually increased worldwide since January 2020 [1]. As of 31 May 2021, there were more than 740,000 confirmed cases of COVID-19 and more than 13,000 COVID-19-related deaths in Japan. Western countries implemented strict lockdowns to prevent the spread of infection and decrease the number of deaths. In contrast, Japan performed case identification, isolation, and contact tracing without strictly enforcing lockdowns [2]. However, there was a daily increase in the number of COVID-19 cases during the first wave during March–April 2021. The national government declared a state of emergency in Tokyo and six prefectures on 7 April 2020, and subsequently extended it nationwide [3]. This was different from lockdown, which was based on regulations followed in other countries and required citizens to voluntarily stay at home to decrease social contact by at least 70%. This strategy was effective in decreasing the number of COVID-19 cases, and the first state of emergency in Japan was lifted on 25 May 2020 [2].

Public health centers (PHCs) play an essential role in infectious disease control in Japan; they are established in each prefecture, designated cities, and core cities [4]. Public health nurses (PHNs) working in PHCs are responsible for conducting active epidemiological surveys, contact tracing, coordinating the hospitalization of COVID-19 patients and monitoring the health of their close contacts, and providing consultation to community residents [5]. However, due to administrative reform in Japan, the number of PHCs have decreased by 40% compared to that in the 1990s [4]. Additionally, asymptomatic patients are required to be in isolation in their homes, accommodations, and hospitals [6]; the capacity of the healthcare system responding to COVID-19 has faced various limitations. PHNs have tried to develop a sustainable healthcare system, such as collaborating with office support staff for telephone consultations, infection control, and outsourcing inventory control [7].

During the pandemic, a telephone consultation system is useful for community residents to obtain general information regarding COVID-19. However, previous studies mainly focused on telemedicine in hospital-based settings [8,9,10]. Only a few studies on COVID-19 were conducted in community-based settings. To reduce infection risk caused by visiting primary healthcare facilities, Oman promoted telephone consultations by physicians [11]. In the UK, cases of mild symptoms and patients consulting for administrative reasons generally use a telephone message system [12]. In Japan, the Ministry of Health, Labour, and Welfare promoted telephone consultation centers for returnees from abroad and people exposed to COVID-19 patients in each PHC, and to provide consultation regarding symptoms such as fever and breathlessness to community residents [13,14]. Thus, community residents seeking consultations regarding COVID-19 infections were directed to the PHCs in each community, and PHNs responded to their concerns. To our knowledge, no studies investigated public telephone consultation systems regarding COVID-19 among community residents. All sectors and research institutes should secure and preserve the records and data regarding COVID-19 to understand the current and future impact on people [15]. Analysis of each consultation is crucial for developing effective and user-friendly systems. Thus, we aimed to (1) describe the characteristics of community residents who used telephone consultation systems, and (2) analyze their concerns during the first wave of COVID-19.

## 2. Materials and Methods

### 2.1. Study Sample

City A is located in the north-central part of Tokyo, with a population of about 230,000, which has been rising in recent years due to the influx of many families; 12% of the population are aged < 18 years, and 19% of the population are aged > 65 years. The elderly population continues to increase yearly, but at a lower rate than the overall aging rate in Japan (28.4%) [16,17]. Although there are some commercial areas in the southern part of the district, City A is primarily residential. Medical institutions of various sizes and functions are located in the city; these range from clinics to university hospitals and public hospitals that provide advanced medical care and having an excellent medical environment in the city is a great advantage.

In February 2020, PHNs established a free telephone consultation hotline regarding COVID-19 for community residents; this service was provided in the disease-prevention section of the PHC. The hotline was open from 9:00 a.m. to 5:00 p.m. every weekday but closed on weekends. When the telephone consultation service was first established, four PHNs were assigned to this section. The disease-prevention section also provides public health services regarding the prevention of tuberculosis and other infectious diseases. PHNs conducted an “active epidemiological survey” for each new positive COVID-19 patient. The “active epidemiological survey” is an investigation conducted mainly by local PHCs in charge of the areas where infectious diseases have occurred. This survey is based on the Japanese Infectious Disease Prevention Act. PHNs conducted this survey to identify individuals who were in close contact with COVID-19 patients; they also cared for patients and monitored their health conditions for 14 days with the objective of preventing the development of new clusters. Additionally, the staff of the disease-prevention section secured hospital beds for positive cases amid a spike in COVID-19 infections and transferred patients to designated hospitals [5]. Typically, in Japan, PHNs are assigned to only one section and do not split any tasks with other sections. However, as the number of telephone consultations increased, the shortage of PHNs became a major issue. Nine PHNs from other departments within the PHC provided support. Simultaneously, the PHC responded by increasing telephone consultation lines from one to four. From 25 March 2020, the PHNs in charge of infection prevention measures in the PHC recorded data on telephone consultation sheets in City A. The PHNs recorded a brief summary of the presented problems and recommendations on these sheets. The residents or organizational representatives initiated the calls analyzed in this study. All the telephone consultation sheets completed between 25 March and 20 April 2020 were analyzed.

### 2.2. Variables on Telephone Consultation Sheets

Telephone consultation sheets included information on the following: (1) consultation date and time; (2) classification of the contacts (“resident” or “organization”), for the organization group, type of organization was assessed (”private companies” or “administrative organizations” or “educational institutions” or “health and welfare related organizations”); (3) age of the contact (categorized as “younger than 20 years old,” “20–29 years old,” “30–39 years old,” “40–49 years old,” “50–59 years old,” “60–69 years old,” “70–79 years old,” or “80 years and older”); (4) sex of the contact; (5) details of the consultation (“having COVID-19-related symptoms” or “having contact with returnees from abroad” or “having contact with a COVID-19 patient ” or “visiting a clinic” or “undergoing polymerized chain reaction (PCR) testing” or “going to work or school” or “other consultations”); (6) recommendations of PHNs resulting from the consultations (“recommendation of primary care doctor visit” or “recommendations for self-care: adequate rest and hydration” or “self-health monitoring” or “provision of information on infection prevention measures” or “provision of COVID-19-related information” or “provision of information on clinics and PCR testing” or “coordination of clinic visits and PCR testing” or “other recommendations”). Multiple answers were allowed for the details of the consultation and recommendations. For analysis of the collected data, we categorized age into two groups, “under 60 years old” or “60 years old and over.” This was based on the results of previous studies [1], which showed that older people have an increased risk for severe COVID-19 infection.

### 2.3. Statistical Analysis

After confirming the distribution of demographic characteristics, time, and estimated duration of each call, we divided the contacts into two groups: community residents were classified into the “resident group” and the organizational representatives of companies, administrative organs, educational institutions, and health- and welfare-related organizations located in City A, were classified into the “organization group.” The term “organizational representatives” refers to individuals who are calling on behalf of their organization. We then analyzed each group according to sex. Chi-squared tests, Fisher’s exact test, and Mann–Whitney U test were conducted to investigate inter-group differences. Statistical significance was set at *p* < 0.05. The data were analyzed using SPSS version 27 (IBM Corp., Armonk, NY, USA).

### 2.4. Ethical Statement

The study was conducted in accordance with the Declaration of Helsinki and was approved by the ethics review board of the University of Tokyo, Japan (No. 2020138NI). This study used anonymized data, therefore written informed consent was not obtained. Furthermore, the Act on the Protection of Personal Information in Japan states that it is unnecessary to obtain informed consent in studies which aim to improve public health and when it is difficult to obtain the individual’s consent.

## 3. Results

### 3.1. Characteristics of COVID-19-Related Free Telephone Consultations

**Figure 1:**
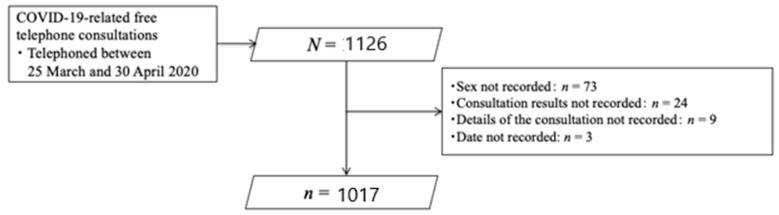
Flowchart of this study.

Of the 1126 telephone consultations recorded by the PHNs, 109 were excluded due to incomplete data (sex, consultation results, consultation details, and date were not recorded in 73, 24, 9, and 3 cases, respectively). Thus, we analyzed 1017 consultations (Figure 1). Table 1 shows the characteristics of the consultations. Of the 1017 patients who received the consultations, 799 (79%) were from the resident group and 218 (21%) were from the organization group. Approximately a quarter of all consultations were provided to people aged 60 years and older. Half of the consultations were provided during midmorning. The average consultation time was 6.8 min (standard deviation (SD) = 4.7).

### 3.2. Characteristics of the Residents Group

Table 2 shows the characteristics of the residents by sex. Among the residents aged ≥ 60 years, the Chi-squared test indicated that there were significantly more females than males. The most common consultation was about “having symptoms” (285 males (83%), and 400 females (88%)). Excluding “other consultations,” the second most frequent consultation was regarding PCR testing (38 males (11%) and 54 females (12%)). The Chi-squared test indicated that a significantly higher percentage of males than females reported “having contact with returnees from abroad.” The most common recommendation of the PHNs was “visiting a primary care doctor” (224 males (65%) and 316 females (69%)). The recommendation of “self-health monitoring” was provided to 92 (27%) males and 119 (26%) females. There were no statistically significant differences in the recommendations of the PHNs according to sex.

### 3.3. Reasons for Seeking Consultation by the Type of Organization

**Figure 2:**
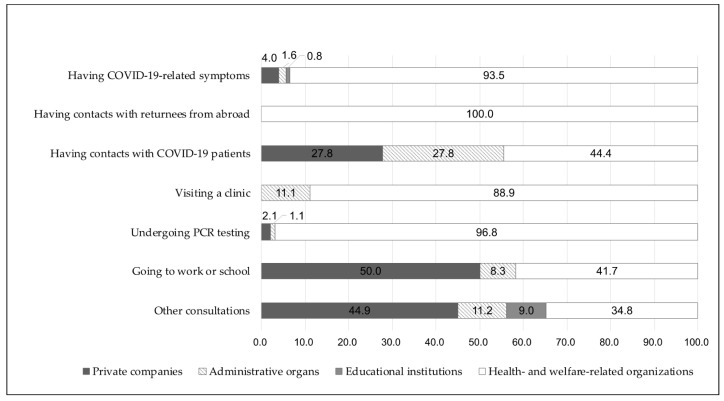
The reasons for calling by the type of organization. Notes: *n* = 218; PCR: polymerized chain reaction.

The consultation details according to each organization type are presented in Figure 2. Health- and welfare-related organizations showed a higher rate of symptom occurrence, contacts with returnees, visits to a clinic, and PCR testing. Private companies showed higher rates of “going to work or school” and “other consultations.”

### 3.4. The Patterns of Both “Consultations Provided to Community People” and “Recommendations of the PHNs for Each Consultation”

In more than 70% of the consultations, PHNs recommended primary care doctor visits for symptomatic consultation, visiting a clinic, and PCR testing (Table 3). The second most common recommendation was self-health monitoring for symptomatic patients or those who had close contacts with COVID-19 patients (including suspected), and for those going to work or school.

## 4. Discussion

To the best of our knowledge, this is the first community-based study focusing on public telephone consultations by PHNs regarding COVID-19 among residents in Japan. We found that approximately 80% of those who received consultations were community residents. Additionally, the results showed that half of the consultations were provided during midmorning. In recent studies, the characteristics of telephone consultations according to the time of day, during COVID-19, have not been clarified [18,19]. Chapman et al. [20] suggested that patient satisfaction with telephone consultations is dependent on the ease of access to health care professionals and the patient-to-telephone line ratio. Therefore, PHCs should ensure a sufficient number of phone lines and staff members to respond to demands of residents adequately.

There was no difference between male and female residents, except that the males had a significantly higher rate of “having contact with returnees from abroad” than the females in terms of consultation content. According to a systematic review and meta-analysis [21] that evaluated gender differences in the prevalence of COVID-19, sex differences were observed in the prevalence of COVID-19. At first, COVID-19 was an unknown infectious disease, and this study was conducted during the first wave of the pandemic in Japan. Thus, the general population experienced anxiety regarding COVID-19 during this period, regardless of their gender.

We found that a high percentage of consultations for health- and welfare-related organizations were related to “having COVID-19-related symptoms” and “PCR testing.” Additionally, for each consultation, the main recommendations of the PHNs were primary care doctor visits and PCR testing. The WHO has recommended that testing all suspected cases is crucial to a pandemic response [22]. In contrast, due to the limited capacity of PCR testing in Japan, PHCs have chosen a cluster-based approach [23]. Only the COVID-19 cases suspected by PHCs were referred to the designated hospitals and clinics to undergo PCR testing [2,5]. To reduce the risk of COVID-19 infection and cross-contamination at medical facilities, the Japanese government recommended that the public should ask before visiting if they have suspected COVID-19 symptoms [24]. This approach prevented residents from rushing to the hospital as a lesson learned from the 2009 H1N1 pandemic [25]. However, it may have confused the decision-making of the staff of health- and welfare- related organizations that received consultations for symptomatic patients and PCR testing. Additionally, in September 2020, the Japanese government promoted telephone consultations with primary care doctors for individuals with suspected COVID-19 symptoms [26]. Thus, local governments should collaborate with primary care physicians and private companies to respond to organizational representatives with consultation requests and ensure an adequate capacity for PCR testing. Furthermore, PHCs should develop a triage system to identify urgent cases among the various telephone consultations. Furthermore, PHCs should develop a new system for responding to an outbreak of infectious diseases and establish more accessible consultation systems for organizational representatives, such as setting up a telephone number, social networking services, and a website.

This study has several limitations. First, this cross-sectional study analyzed only one local government and a limited number of free telephone consultation data which included missing data. Second, we could not conduct a power analysis in this study. Third, we could not identify a causal relationship in this cross-sectional study. Fourth, “other consultations” accounted for approximately 18% of all consultations; however, we were unable to analyze the details of this category. Fifth, in principle, “organizational representatives” refer to individuals who are calling on behalf of their organization; however, we cannot exclude the possibility that it includes consultation regarding one’s concerns as a resident. In Japan, personal health consultations regarding COVID-19 are discussed with the local government, where the individual lives as a resident. Thus, the consultations for the “organization group” would include consultation as a “resident.” The generalization of the results may be limited to other municipalities in Japan. Future research should collect more data to reveal the consulters’ characteristics regarding COVID-19 in the community and develop more effective health communication measures to understand COVID-19 and prevent its spread.

## 5. Conclusions

We analyzed 1017 public telephone consultations by PHNs, of which approximately 80% were provided to community residents and 20% to organizational representatives located in City A. The most common consultation was about having COVID-19-related symptoms. The results suggest that PHCs should provide more helpful information on COVID-19 that considers concerns regarding the characteristics of the consultors. To combat COVID-19 and support community residents, PHCs and PHNs should improve the healthcare system more effectively and efficiently based on the current evidence.

## Figures and Tables

**Table 1:** Characteristics of COVID-19-related free telephone consultations (*N* = 1017).

Variables	Total (*N* = 1017) *N* (%) or Mean ± SD (Min–Max)	The Resident Group (*n* = 799) *n* (%) or Mean ± SD (Min–Max)	The Organization Group (*n* = 218) *n* (%) or Mean ± SD (Min–Max)	*p*
Sex				
Male	471 (46.3)	343 (42.9)	128 (58.7)	<0.001 *
Age (*n* = 662)				
<60 years old	498 (75.2)	408 (76.7)	90 (69.2)	0.077 *
≥60 years	164 (24.8)	124 (23.3)	40 (30.8)	
Consultation time				
9 a.m. to 11 a.m.	508 (50.0)	387 (48.4)	121 (55.5)	0.045 *
12 p.m. to 14 p.m.	261 (25.7)	219 (27.4)	42 (19.3)	
15 p.m. to 17 p.m.	248 (24.4)	193 (24.2)	55 (25.2)	
Duration of consultation: min (*n* = 622)	6.8 ± 4.7 (1–50)	6.9 ± 4.8 (1–50)	6.6 ± 4.1 (1–20)	0.716 ^§^
Categories of organizations				
Private companies	-	-	47 (21.6)	-
Administrative organs	-	-	14 (6.4)	
Educational institutions	-	-	9 (4.1)	
Health- and welfare-related organizations	-	-	148 (67.9)	

Notes: Categories of organizations private companies, administrative organs, educational institutions, and health- and welfare-related organizations in City A; SD: standard deviation; *p*: *p*-value (2-tailed), min: minutes; * = Chi-squared test, § = Mann–Whitney U test.

**Table 2:** Characteristics of consultations by the sex differences in the resident group (*N* = 799).

Variables	Male (*n* = 343) *n* (%) or Mean ± SD (Min–Max)	Female (*n* = 456) *n* (%) or Mean ± SD (Min–Max)	*p*
Age (*n* = 532)			
<60 years old	182 (81.6)	226 (73.1)	0.023 *
≥60 years old	41 (18.4)	83 (26.9)	
Consultation time			
9 a.m. to 11 a.m.	172 (50.1)	215 (47.1)	0.644 *
12 p.m. to 14 p.m.	93 (27.1)	126 (27.6)	
15 p.m. to 17 p.m.	78 (22.7)	115 (25.2)	
Duration of consultation: min	6.9 ± 95.0 [1–35]	6.9 ± 94.7 [1–50]	0.538 ^§^
Consultation details: multiple answer			
Having COVID-19-related symptoms	285 (83.1)	400 (87.7)	0.064 *
Having contacts with returnees from abroad	24 (7.0)	16 (3.5)	0.025 *
Having contacts with COVID-19 patients	27 (7.9)	34 (7.5)	0.827 *
Visiting a clinic	25 (7.3)	39 (8.6)	0.515 *
Undergoing PCR testing	38 (11.1)	54 (11.8)	0.738 *
Going to work or school	20 (5.8)	21 (4.6)	0.437 *
Other consultations	48 (14.0)	51 (11.2)	0.233 *
Recommendations of PHNs for the consultations: multiple answer			
Recommendation of primary care doctor visit	224 (65.3)	316 (69.3)	0.233 *
Recommendations for self-care: adequate rest and hydration	55 (16.0)	68 (14.9)	0.663 *
Self-health monitoring	92 (26.8)	119 (26.1)	0.818 *
Provision of information on infection prevention measures	36 (10.5)	48 (10.5)	0.989 *
Provision of COVID-19 information	32 (9.3)	33 (7.2)	0.284 *
Provision of information on clinics and PCR testing	22 (6.4)	24 (5.3)	0.489 *
Coordination of clinic visits and PCR testing	10 (2.9)	21 (4.6)	0.221 *
Other recommendations	4 (1.2)	4 (0.9)	0.685 *

Notes: PCR: polymerized chain reaction; PHN: public health nurses; SD: standard deviation; *p*: *p*-value (2-tailed); min: minutes; * = Chi-squared test, § = Mann–Whitney U test.

**Table 3:** The proportion of PHNs’ recommendations by each consultation (*N* = 1017).

Recommendations of PHNs for Each Consultation	Consultation Details from Community People						
	Having COVID-19-Related Symptoms (*n* = 809)	Having Contacts with Returnees from Abroad (*n* = 42)	Having Contacts with COVID-19 Patients (*n* = 79)	Visiting a Clinic (*n* = 73)	Undergoing PCR Testing (*n* = 187)	Going to Work or School (*n* = 53)	Other Consultations (*n* = 188)
Recommendation of primary care doctor visit	623 (77.0)	27 (64.3)	28 (35.4)	52 (71.2)	137 (73.3)	11 (20.8)	24 (12.8)
Recommendations for self-care: adequate rest and hydration	122 (15.1)	5 (11.9)	8 (10.1)	3 (4.1)	9 (4.8)	3 (5.7)	8 (4.3)
Self-health monitoring	183 (22.6)	15 (35.7)	24 (30.4)	6 (8.2)	17 (9.1)	22 (41.5)	29 (15.4)
Provision of information on infection prevention measures	67 (8.3)	3 (7.1)	18 (22.8)	5 (6.8)	7 (3.7)	9 (17.0)	53 (28.2)
Provision of COVID-19-related information	30 (3.7)	4 (9.5)	17 (21.5)	6 (8.2)	10 (5.3)	14 (26.4)	85 (45.2)
Provision of information on clinics and PCR testing	32 (4.0)	0 (0.0)	3 (3.8)	8 (11.0)	29 (15.5)	4 (7.5)	26 (13.8)
Coordination of clinic visits and PCR testing	119 (14.7)	2 (4.8)	10 (12.7)	7 (9.6)	91 (48.7)	1 (1.9)	4 (2.1)
Other recommendations	8 (1.0)	0 (0.0)	0 (0.0)	1 (1.4)	2 (1.1)	0 (0.0)	16 (8.5)

Notes: *n* = 1017; PCR: polymerized chain reaction; PHN: public health nurses.

## Data Availability

The data presented in this study are not publicly available because of privacy restrictions.
